# Shaping ability of WaveOne Gold and OneReci by using two apical sizes: a micro-computed tomographic assessment

**DOI:** 10.7717/peerj.15208

**Published:** 2023-04-27

**Authors:** Elif Çiftçioğlu, Ali Keleş, Gözde Akbal Dinçer, Melis Oya Ateş, Enver Sedat Küçükay

**Affiliations:** 1 İstanbul Okan University, İstanbul, Turkey; 2 Abant Izzet Baysal University, Bolu, Turkey; 3 Ondokuz Mayis University, Samsun, Turkey

**Keywords:** Shaping ability, OneReci, WaveOne Gold, Micro-computed tomography, Apical enlargement

## Abstract

**Backround:**

OneReci (MicroMega, Besançon, France) is a recently introduced single-file reciprocating system with scarce information revealed on its shaping ability. This study aimed to compare the shaping abilities of OneReci and a well-documented single-file reciprocating system WaveOne Gold (WOG; Dentsply Maillefer, Ballaigues, Switzerland) and evaluate the effect of increased apical enlargement on the preparation quality, using micro-computed tomography (micro-CT).

**Methods:**

After an initial micro-CT scanning, twenty mesial root canals of mandibular molars were anatomically matched. The canals were assigned to two experimental groups (*n* = 10), using OneReci or WOG in different canals of the same root. The glide paths were created, and root canals were prepared twice, using size 25 and 35 instruments of the systems, respectively. The specimens were scanned with micro-CT after each preparation. The increase in canal volume, amount of dentin removal, unprepared root canal surface, canal transportation, centering ratio and preparation times were assessed. The data were analysed with independent sample *t*-tests, variance analyses, Friedman and Mann-Whitney U tests. The significance level was set at 5%.

**Results:**

Each preparation increased the canal volume and dentin removal while decreasing the unprepared root surface. The difference between the systems became significant after preparation with size 35 instruments (*p* < 0.05). Regarding canal transportation and centering ratio, the difference was insignificant (*p* > 0.05). The first preparation step (glide path + size 25 instrument) was significantly faster in the OneReci group (*p* < 0.05).

**Conclusions:**

Preparation with size 25 instruments of the systems appeared to be safe with similar shaping performances. Larger apical preparation promoted significantly higher dentin removal, volume increase, and prepared surface area in WOG.

## Introduction

The mechanical objectives of root canal instrumentation are well established as the maintenance of the original canal anatomy with centered instrumentation, reducing preparation errors and preserving the structural integrity by retaining root dentin as much as possible (Bürklein & Schafer, 2013; Arias & Peters, 2022). The quality and efficiency of mechanical instrumentation have greatly improved after the introduction of automated nickel-titanium (Ni-Ti) systems and with the implemented variations to optimize their mechanical properties over time, such as design and kinematics of the instruments as well as surface and heat treatment procedures (Zupanc, Vahdat-Pajouh & Schäfer, 2018; Velozo et al., 2020).

WaveOne Gold (WOG; Dentsply Maillefer, Ballaigues, Switzerland) is one of the well-positioned reciprocating single-file systems in the market. WOG files have an alternating off-centered parallelogram-shaped cross-section and undergo a special thermal treatment (Ruddle, 2016; Kharouf et al., 2022). These design features of WOG files are suggested to improve flexibility while causing less screw-effect and apical transportation (Ruddle, 2016). Four sizes of WOG are available in the market: Small (20.07), Primary (25.07), Medium (35.06), and Large (45.05).

However, even with currently available Ni-Ti systems, studies have shown evidence of unprepared root canal surfaces, a certain amount of canal transportation, and decentralization (Da Silva Limoeiro et al., 2016; Versiani et al., 2018; Pérez et al., 2018; Siqueira Jr et al., 2018; Stringheta et al., 2019; Duque et al., 2019; Perez Morales et al., 2020; Haupt, Pult & Hülsmann, 2020). Among the several methodologies evaluating the shaping ability of instruments, micro-computed tomography (micro-CT) is considered as the gold standard (Versiani et al., 2018; Hülsmann, 2022). High-resolution micro-CT technology is a non-invasive and a non-destructive method (Siqueira Jr et al., 2018; Hülsmann, 2022). This technique allows a three-dimensional investigation of extracted teeth regarding changes in the canal volume, anatomy, and extent of unprepared root canal surface by comparing pre-and post-operative root canal morphology (Siqueira Jr et al., 2018; Stringheta et al., 2019; Duque et al., 2019; Perez Morales et al., 2020; Hülsmann, 2022).

Recently a new reciprocating single-file system, OneReci (MicroMega, Besançon, France), was introduced on the market. OneReci files are manufactured from a 1 mm diameter wire and are featured a heat treatment (C-wire) with an asymmetric cross-sectional design that changes to an S-shape towards the shank (Seracchiani et al., 2022; Kharouf et al., 2022). OneReci is available in five sizes: 20.04, 25.04, 25.06, 35.04, and 45.04. The manufacturer claims that OneReci files respect the root canal anatomy with a minimally invasive and centered preparation.

Before root canal shaping, creating a glide path is recommended (Bürklein & Schafer, 2013; Ruddle, 2016). Glide path preparation with automated Ni-Ti files produces less transportation and preserves the canal anatomy better than K-files (Bürklein & Schafer, 2013; Vorster, Van der Vyver & Paleker, 2018; Van der Vyver et al., 2019). One G (OG; MicroMega, Besançon, France) and WaveOne Gold Glider (WOGG; Dentsply Maillefer, Ballaigues, Switzerland) are the mechanical glide path preparation systems of OneReci and WOG, respectively (Kharouf et al., 2022; Van der Vyver et al., 2019; Vorster, Van der Vyver & Paleker, 2018).

The safe clinical usage of any Ni-Ti instrument requires understanding its correlation with root canal anatomy as well as the applied technologies and mechanical properties (Peters, 2004). In this respect, WOG could be considered a well-documented system (Seracchiani et al., 2022; Stringheta et al., 2019; Duque et al., 2019; Haupt, Pult & Hülsmann, 2020; Garcia et al., 2019; Kharouf et al., 2022; Vorster, Van der Vyver & Paleker, 2018). Nevertheless, the data on debris extrusion (Kharouf et al., 2022), mechanical performance, and metallurgical characteristics (Seracchiani et al., 2022) of OneReci have been demonstrated. However, regarding the shaping ability of OneReci, there is limited information provided in a recently published cone-beam computed tomography (CBCT) study (Mann et al., 2022).

Therefore, this *ex vivo* study aimed to compare the shaping ability of two reciprocating single-file systems, OneReci and its well-known counterpart WOG, and assess the effect of increased apical enlargement on the preparation quality through micro-CT analysis.

The null hypothesis was that there were no significant differences between WOG and OneReci with regard to the amount of dentin removal, root canal volume increase, the percentage of unprepared root canal surface, root canal transportation, centering ability, and preparation time.

## Material and Methods

The study protocol was approved by the İstanbul Okan University Research Ethics Committee (146/2021).

### Sample selection

Based on a previous study (Velozo et al., 2020) comparing the shaping ability of two different systems, for an effect size of 1.296 and providing a test power of 0.80 with an alpha-type error probability of 0.05, the minimum study sample size to obtain statistical validity was calculated as nine specimens per group by using a priori type power analysis (G*Power 3.1.9.6, Heinrich Heine, Universität Düsseldorf).

From a pool of mandibular molars, forty-five mandibular first molars with fully formed apices were selected and stored in a 0.1% thymol solution at 4 °C for disinfection. After performing coronal accesses, size 10 K-files (Dentsply Maillefer, Ballaigues, Switzerland) were inserted into the mesial root canals, and radiographs were taken from the buccolingual and mesiodistal directions. Consequently, only fifteen molars were identified with two separate mesial canals and foramina, and an initial foramen diameter coinciding with a size 10 K-file. Distal roots of these teeth were sectioned with a diamond disc and discarded. The occlusal surfaces of the specimens were ground with a diamond bur, and the side of the mesiobuccal canal was marked by creating a slight groove in the coronal aspect. The fifteen specimens were then numbered and kept in distilled water-filled bottles throughout the experiment. 

After the initial micro-CT scanning (Skyscan 1172; Bruker-microCT, Kontich, Belgium), ten of the specimens that had type IV root canals (Vertucci, 1984) and moderate curvatures ranging between 10°–30°, according to the Schneider method (Schneider, 1971), were selected for further experimental procedures.

Afterward, the mesiobuccal and mesiolingual root canals (*n* = 20) were matched based on their morphological features and allocated to one of the experimental groups (*n* = 10) according to the preparation systems. Aiming to optimize the standardization of the groups, either OneReci or WOG was used in each of the matched root canals of the same specimen, allowing each instrument to be used in five mesiobuccal and five mesiolingual canals. The normality assumption was verified, and the homogeneity of the groups regarding curvature degree, surface area, and volume of the specimens was statistically confirmed at baseline (*p* > 0.05; Table 1).

**Table 1:** Baseline mean and standard deviation values of degree of curvature, canal volume and surface area for the preparation systems. The same superscript letters on the columns indicate no statistically significant difference (*p* > 0.05).

**System**	**Degree of curvature (°)**	Volume (mm^3^)	Surface area (mm^2^)
WaveOne Gold	21.83 ± 6.8^a^	1.07 ± 0.43^a^	15.87 ± 4.98^a^
OneReci	22.83 ± 6.12^a^	1.16 ± 0.54^a^	15.43 ± 5.49^a^
*p*-value	0.734	0.838	0.852

### Root canal preparation protocols

A size 10 K-file was inserted into the mesiobuccal and mesiolingual canals until its tip was visible through the apical foramen under a dental microscope (Leica M320; Leica Microsystems, Wetzlar, Germany). The working length (WL) of both canals was established as one mm short of this measure.

The specimens were initially fixed on a jaw model (Frasaco GmbH, Tettnang, Germany) at their anatomical positions and mounted on a phantom head (KaVo, NC, USA) to create a realistic setting (Hülsmann, 2022).

All canal preparations were performed under rubber-dam (Hygienic Dental Dam, Coltene Whaledent Inc., Germany) isolation by a single endodontist experienced in using tested systems to avoid operator-related variables. Each glide path and shaping file were used only once per root canal.

In the OneReci group, the glide paths were created using an OG at the WL, with an endodontic motor (Dual Move; MicroMega, Besançon, France) operated at 300 rpm and 1.2 N. The root canals were then prepared with OneReci 25.04, which is recommended for root canals with an initial canal diameter corresponding to a size 10 instrument. The instrument was used until the WL was reached in a reciprocating motion (170 CCW, 60 CW) of the Dual Move motor. After root canal preparation, the specimens were scanned by micro-CT. For the second preparation assessment, the root canals were then prepared using OneReci 35.04 in the same manner.

In the WOG group, the glide paths were created using WOGG at WL in reciprocating motion operated at the “WaveOne Gold” mode of the X-Smart Plus endodontic motor (Dentsply Maillefer, Ballaigues, Switzerland). The root canals were then prepared with WOG Primary instrument (25.07) until the WL was reached using the same mode of X-Smart Plus. After micro-CT scanning, the root canals were prepared using WOG Medium (35.06) and operated similarly.

Once the preparation procedures were completed, the specimens were subjected to a final micro-CT scanning. Therefore, three micro-CT scans were performed per specimen.

All root canal preparations were carried out following the techniques prescribed by the manufacturers. After each passage, the root canals were irrigated with 2 mL of 5% sodium hypochlorite (NaOCl) using 30-G side-vented needles (ProRinse; Dentsply Maillefer, Ballaigues, Switzerland), recapitulated with a size 10 K-file, and the instruments were cleaned with gauze impregnated to 70% isopropyl alcohol. These procedures were repeated until the WL was achieved. A final irrigation was performed with 5 mL of 17% EDTA followed by 5 mL of 5% NaOCl, and the root canals were dried using paper points (Tosco et al., 2023). Prior to micro-CT scans, the outer apical surfaces of the specimens were brushed and rinsed to remove potential extruded debris.

### Micro-CT scanning procedures

Each specimen was placed on the rotating platform of the micro-CT device (Skyscan 1172; Bruker micro-CT, Kontich, Belgium) and individually scanned using the following parameters: voltage of 80 kV, current of 124 µA, pixel size of 13.68 µm, rotation range of 180° and step of 0.5°, exposure time of 2,400 ms, and frame average of two with a 1 mm-thick aluminium+cupper filter. The projection images were reconstructed with 75% beam hardening correction, three smoothing, and nine ring artifact correction using the NRecon software (v1.6.10.6, Bruker micro-CT). All scans were performed in the same position using the same parameters as the initial one to ensure the accuracy of the subsequent analysis.

The pre-and postoperative images were then superimposed by using the 3D registration function of the DataViewer v.1.5.1 (Bruker microCT) and processed in CTAn v.1.14.4 (Bruker microCT) to calculate quantitative parameters and build visual 3D models (Da Silva Limoeiro et al., 2016; Perez Morales et al., 2020). The following parameters were analysed: increase in the root canal volume, unprepared root canal surface, amount of dentin removal, canal transportation, and centering ability. CTVol 2.3.2.0 software (Bruker-microCT) was used for qualitative evaluation of shaping ability of the tested systems. A color-code was defined for root canal models using yellow for the unprepared root canals, dark blue for the 1st, and light blue for the 2nd preparations.

All analyses were performed by an examiner blinded to the preparation protocols.

### Evaluation of the parameters

In this study, the percentage of increase in the root canal volume (VI%) after each preparation step was calculated using the values for the unprepared root canal (X), and after the first (Y) and the second (Z) preparations of the root canals, based on the formula (Versiani et al., 2018): VI% = [(Y or Z] − X) / X * 100.

The amount of dentin removal was calculated by subtracting the values of prepared root canals from the unprepared counterparts (Da Silva Limoeiro et al., 2016; Perez Morales et al., 2020).

Matched images of the surface areas before and after preparations of the root canals were examined, and the percentage of the unprepared root canal surface areas was calculated based on the ratio of static voxels to the total number of voxels on the canal surface (Da Silva Limoeiro et al., 2016; Perez Morales et al., 2020; Haupt, Pult & Hülsmann, 2020).

The canal transportation and centering ratio assessments were performed on the axial sections at 3, 6, and 9 mm distances from the anatomic apex (Perez Morales et al., 2020). The shortest distance from the outer surface of the root to the outer surface of the unprepared root canal, to the root canal surface after the first preparation and the second preparation was measured in both mesial and distal directions following the method of Gambill, Alder & Carlos (1996). The obtained values in mm were then applied to the formulas described in the aforementioned study (Gambill, Alder & Carlos, 1996). According to the transportation formula, a result of “0” indicated no canal transportation. A negative value pointed to canal transportation toward the inner curvature and a positive value to the outer curvature. For centering ratio evaluation, a result of “1” indicated an optimum centering ability.

The active working time of each instrument in the root canal was measured with an electronic stopwatch and recorded as preparation time. The time taken to clean the flutes of the instruments and irrigation were not taken into account.

### Statistical analysis

Preoperatively, the data regarding the morphological features of the specimens were checked for normality of distribution with the Shapiro–Wilk test, and group homogeneity was affirmed with independent sample *t*-tests (Table 1).

The normally distributed data were analysed with independent sample t-tests and variance analyses. Friedman and Mann–Whitney U tests were used when the normality was rejected. The chi-square test was used to determine the intragroup and intergroup differences in the percentages of the transportation direction. The mean ± standard deviation was given as descriptive statistics. All data analyses were performed using SPSS software v.23.0 (IBM SPSS, Inc., Chicago, IL, USA) with a significance level set at 5% (*p* < 0.05).

## Results

Table 1 shows the homogeneous distribution of the file systems among the specimens in terms of curvature degree, canal volume, and surface area. No instrument fractures or procedural errors occurred during root canal preparation.

Each preparation step generally increased the canal volume and dentin removal in both groups while decreasing the unprepared root canal surface. After both preparations, the overall mean values of dentin removal, the percentage of increase in canal volume, and the decrease in unprepared root canal surface were higher in the WOG group. However, the difference between the systems was significant only after the second preparation (Table 2).

**Table 2:** Mean and standard deviation values for dentin removal, canal volume increase and untouched root canal surface after each preparation step. Different superscript letters on the same column indicate a statistically significant difference (*p* < 0.05).

**Step**	**System**	**Dentin Removal**	**Canal Volume Increase (%)**	**Untouched Root Canal Surface (%)**
After First Preparation	WaveOne Gold	0.93 ± 0.67	102.99 ± 78.4	21.319 ± 9.73
	OneReci	0.462 ± 0.35	48.34 ± 41.2	28.025 ± 6.91
	*p*-value	0.067	0.072	0.092
After Second Preparation	WaveOne Gold	1.845 ± 0.65^a^	196.12 ± 90.1^a^	10.905 ± 4.28^a^
	OneReci	0.916 ± 0.37^b^	95.88 ± 47.5^b^	19.320 ± 6.22^b^
	*p*-value	0.001	0.008	0.002

**Figure 1:**
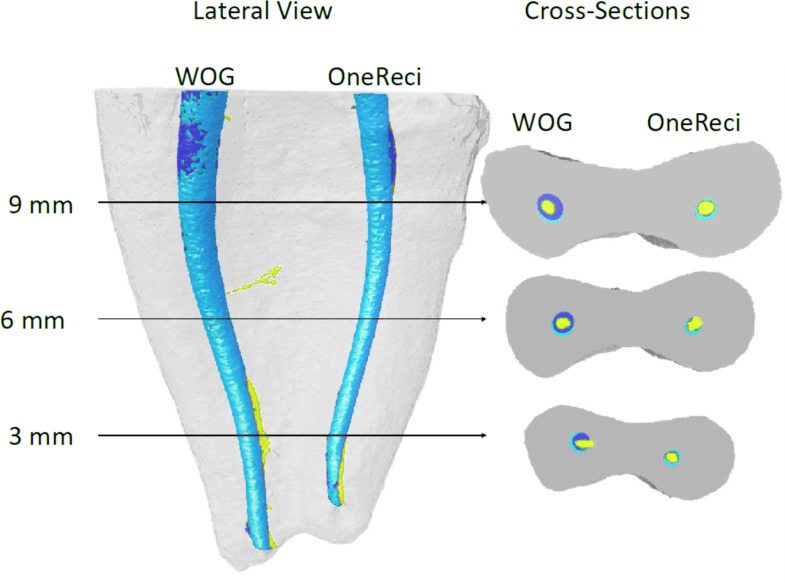
A lateral view of the representative three-dimensional reconstruction of the internal anatomy of the superimposed mesial root canals before (yellow), after first preparation (dark blue), and second preparation (light blue). Representative cross-sections of the superimposed root canals with WOG and OneReci, at the 3, 6, and 9 mm.

The difference between OneReci and WOG was insignificant regarding canal transportation and centering ratio at all three levels (3, 6, and 9 mm) (*p* > 0.05). The transportation values increased from 3 mm to 9 mm without any significant difference (*p* > 0.05). Table 3 shows the mean canal transportation and centering ratio values with the percentage of transportation direction at evaluated levels. The representative image (Fig. 1) depicts the axial canal changes after each canal preparation with WOG and OneReci.

**Table 3:** The mean (± standard deviation) transportation and centering ratio for WaveOne Gold and OneReci at different apical levels after each preparation. Superscript letters indicate statistically significant differences (*p* < 0.05). Lowercase letters show the difference among the levels and uppercase letters between the systems.

		**WaveOne Gold**			**OneReci**		
		**3 mm**	**6 mm**	**9 mm**	**3 mm**	**6 mm**	**9 mm**
First Preparation	Mean Transportation (mm)	0.03 ± 0.04	0.10 ± 0.12	0.14 ± 0.12	0.04 ± 0.03	0.06 ± 0.11	0.07 ± 0.07
	Transportation towards inner curvature (%)	%60^a^	%70^b^	%90^b^	%70	%70	%70
	Centering ratio	0.41 ± 0.29	0.32 ± 0.38	0.27 ± 0.26	0.28 ± 0.24	0.42 ± 0.36	0.40 ± 0.38
Second Preparation	Mean Transportation (mm)	0.11 ± 0.24	0.11 ± 0.10	0.14 ± 0.13	0.03 ± 0.03	0.05 ± 0.04	0.08 ± 0.07
	Transportation towards inner curvature (%)	%20^Aa^	%60^Ab^	%80^b^	%40^Ba^	%100^Bb^	%90^b^
	Centering ratio	0.58 ± 0.27	0.48 ± 0.30	0.46 ± 0.34	0.78 ± 0.20	0.33 ± 0.24	0.42 ± 0.34

When the preparation times were compared, the glide path preparation with OG (6.73 ± 1.5 s) showed a significantly shorter mean preparation time than WOGG (9.39 ± 2.1 s) (*p* = 0.005). There was no significant difference between the two file systems regarding preparation times of size 25 and size 35 instruments (*p* = 0.098 and *p* = 0.569, respectively). However, the mean first preparation step (glide path+size 25 instrument) was significantly faster in the OneReci group (*p* = 0.049) (Table 4).

**Table 4:** Preparation times (s) of the systems. Different superscript letters on the same column indicate a statistically significant difference (*p* < 0.05).

**System**	**Glide path files**	**Size 25 files**	**First Preparation** **(Glide path+size 25 files)**	**Second Preparation** **Size 35 files**
WaveOne Gold	9.39 ± 2.1^a^	23.4 ± 9.3^a^	32.79 ± 10.8^a^	17.23 ± 6.2^a^
OneReci	6.73 ± 1.5^b^	16.14 ± 9.4^a^	22.86 ± 10.2^b^	19.65 ± 11.7^a^
*p*-value	0.005	0.098	0.049	0.569

## Discussion

In the current study, two heat-treated reciprocating single-file systems, OneReci and WOG, were compared regarding their preparation effectiveness on moderately curved mesial roots of mandibular molars. This is the first micro-CT study evaluating the shaping ability of OneReci. In the meantime, the influence of increased apical size on the preparation quality was also assessed by using the larger instruments of the systems. Despite the different manufacturing characteristics of selected instruments except for their apical sizes, after the first preparation step (glide path + size 25 instruments), the results indicated a comparable shaping ability (Tables 2 and 3). However, preparation with size 35 instruments demonstrated significant differences between the systems regarding dentin removal, volume increase, and unprepared root canal surface. Therefore, the null hypothesis was partially rejected.

The shaping performance of the instruments was mainly based on the evaluation of various parameters, including the untouched root canal surface, dentin removal, the increase in root canal volume, canal transportation, centering ability, and preparation time (Velozo et al., 2020; Da Silva Limoeiro et al., 2016; Versiani et al., 2018; Pérez et al., 2018; Duque et al., 2019; Haupt, Pult & Hülsmann, 2020). In the present study, all mentioned parameters were precisely evaluated with the advantage of a 3D comparison of pre-and post-preparation image analysis and the non-destructive structure of micro-CT (Da Silva Limoeiro et al., 2016; Versiani et al., 2018; Pérez et al., 2018; Duque et al., 2019; Perez Morales et al., 2020; Haupt, Pult & Hülsmann, 2020; Hülsmann, 2022).

In comparative studies, the well-balanced groups with anatomical matching and standardization allow smaller sample sizes without compromising the reliability of the results (De-Deus et al., 2020). The specimens of the current study were meticulously selected from micro-CT scanned type IV (Vertucci, 1984) mesial root canals of mandibular molars, and the baseline similarity regarding morphological features of the groups was verified to ensure the reliability of methodological comparison and eliminate the teeth-related variables (Velozo et al., 2020; Versiani et al., 2018; Stringheta et al., 2019) (Table 1). This selection allowed the evaluation of two preparation systems on matched samples with similar anatomies. In addition, to create a realistic clinical setting, the systems were tested on the specimens fixed to the jaw model according to their anatomical positions that were mounted on a phantom head (Hülsmann, 2022).

Despite all well-intentioned attempts to improve the shaping ability of instruments, micro-CT studies have conclusively revealed the presence of unprepared areas in the root canal (Paqué, Ganahl & Peters, 2009; Velozo et al., 2020; Da Silva Limoeiro et al., 2016; Pérez et al., 2018; Siqueira Jr et al., 2018; Stringheta et al., 2019; Duque et al., 2019; Perez Morales et al., 2020; Haupt, Pult & Hülsmann, 2020). These areas may harbor bacterial biofilms and tissue remnants that could act as a potential cause of reinfection (Siqueira Jr et al., 2018). Clinically, determining an optimum preparation size that maintains the balance between adequate disinfection and structural integrity of the root canal is challenging (Peters, 2004; Paqué, Ganahl & Peters, 2009). Small preparation sizes are recommended to avoid transportation and unnecessary dentin removal (Paqué, Ganahl & Peters, 2009; ElAyouti et al., 2011). On the other hand, the larger apical preparation promotes bacterial reduction, improves canal disinfection, and decreases the unprepared root canal surface (Pérez et al., 2018; Duque et al., 2019; Rodrigues et al., 2017).

In the literature, the mean unprepared surface area reported for WOG Primary ranged between 16.44–28.67% (Stringheta et al., 2019; Duque et al., 2019; Haupt, Pult & Hülsmann, 2020). This ratio was between 20–34% for OneReci in the sections taken from different root canal levels (Mann et al., 2022). Consistent with these values, after preparation with WOG Primary and OneReci (25.04), our study demonstrated that 21.3% and 28% of the canal surface remained unprepared, respectively.

The amount of unprepared root canal surface was correlated with apical canal size (Paqué, Ganahl & Peters, 2009) and shown to be reduced after larger preparations (Pérez et al., 2018; Duque et al., 2019). This is in line with our observations indicating less unprepared root surfaces after preparation with OneReci (35.04) and WOG Medium files (Table 2). Despite the similar apical sizes of 35, the difference between the systems was significant (*p* = 0.002). This can be attributed to the small taper of the OneReci, which is more prominent in the coronal part due to the use of a 1 mm diameter wire to optimize the dimension of the instrument (Seracchiani et al., 2022).

As demonstrated before, the canal volume and dentin removal were increased after each preparation step (Pérez et al., 2018; Duque et al., 2019). However, the amount of increase is a matter to be approached with caution because excessive dentin removal jeopardizes the integrity of the tooth and leads to vertical root fracture (Ossareh, Rosentritt & Kishen, 2018). Although the values were higher in the WOG group, the difference was insignificant after the first preparation. On the other hand, increasing the instrument size yielded significant differences between OneReci and WOG. Duque et al. (2019) also reported a significant volume increase after the second preparation with a larger WOG (35.06) instrument. Since this is the first micro-CT study demonstrating the volume increase of OneReci, a direct comparison of the data was not possible. However, the difference between OneReci and WOG can be attributed to the smaller cross-sectional area of OneReci and the greater taper of WOG instruments (Seracchiani et al., 2022). The insignificant differences between the systems when using smaller instruments can be assumed to become more pronounced during preparation with larger sizes, leading to a significant difference.

Regarding canal transportation and centering ability, the present study demonstrated no significant difference between the systems at any of the evaluated levels (Table 3). Irrespective of the system, optimum centering could not be achieved. Compared to WOG, the more flexible nature of OneReci with a reduced core diameter and taper (Seracchiani et al., 2022) may have resulted in small transportation values. However, considering the similar centering ratio values, the unique asymmetric cross-sectional design of OneReci does not seem to contribute to superior centering ability.

The amount of transportation tended to increase coronally, parallel with the increment in the cross-sectional diameter of the instruments. High transportation values at the coronal level indicate more peri-cervical dentin loss that strongly affects the long-term survival of the tooth (Clark & Khademi, 2010). However, within all sections, the mean transportation ranged between 0.03 and 0.14 mm (Table 3), which could be negligible compared to the suggested critical apical level of 0.3 mm (Wu et al., 2000). Therefore, both systems appeared to maintain the original root path, even at larger apical sizes. These outcomes corroborate previous studies that evaluated canal transportation and the centering ability of WOG (Duque et al., 2019; Vorster, Van der Vyver & Paleker, 2018) and OneReci (Mann et al., 2022).

The study results presented a transportation most frequently directed towards the inner curvature, except for the apical sections of the roots after larger preparations (Table 3). The inward direction of apical canal transportation was explained by the low stiffness and high flexibility of the instruments (Kaptan et al., 2005), which may change with an increase in the size, taper, and core diameter (Bürklein & Schafer, 2013). Based on this assumption, size 35 instruments could promote more mesial transportation in the apical sections.

However, our results contradict early investigations that showed OneReci (Mann et al., 2022) and at 3 mm WOG Primary (Duque et al., 2019; Haupt, Pult & Hülsmann, 2020), mainly transports the canal through the outer curvature. The discrepancy between the studies is probably due to different experimental models and instrumentation techniques. In the present study, the systems were tested on the specimens mounted on a phantom head to simulate actual clinical conditions (Hülsmann, 2022). However, in this set-up, the positions of the teeth in the arch, the presence of antagonist upper teeth and limited mouth opening might restrict the perpendicular approach and required a slight angulation of the instruments for straight-line access. Another important difference between the studies was creating a glide path with OG and WOGG before using size 25 instruments. Besides superelastic Ni-Ti metal properties, increased flexibility of WOGG (Van der Vyver et al., 2019) and high centering ability of OG (Vorster, Van der Vyver & Paleker, 2018) may have contributed to preserving the original canal shape without any outward transportation. Furthermore, our experiments were carried out at room temperature, where both systems exhibited martensitic characteristics (Seracchiani et al., 2022; Garcia et al., 2019), thus being more flexible with the advantage of pre-bending (Zupanc, Vahdat-Pajouh & Schäfer, 2018). The demonstrated high austenite finish (Af) temperatures of OneReci (41.40° ± 0.10 °C) (Seracchiani et al., 2022) and WOG (49.96° ± 0.04 °C and 48.9° ± 2.2 °C) (Seracchiani et al., 2022; Garcia et al., 2019) could also be interpreted as the acquired shape of the instruments in the canal could maintain unchanged and might have driven the instruments towards the inner curvature.

Preparation times of OneReci and WOG were identical when size 25 and size 35 instruments were used. However, the glide path preparation times were statistically different between OG and WOGG (*p* = 0.005). Therefore, the first canal preparation step (glide path+ size 25 instrument) was significantly faster with OneReci due to the short glide path preparation times of OG (Table 4). A recent study (Pillay, Vorster & Van der Vyver, 2021) showed significantly faster preparation times for WOGG than OG, which differs from our findings. This might be caused by the plastic training block usage in the latter study since cutting efficiency and preparation times of files on plastic, and dentin could change due to differences in hardness (Hülsmann, 2022).

From a clinical point of view, all parameters evaluated in this study have an impact on the treatment outcome. Therefore, proper instrument selection for root canal preparation gains more importance considering each system has its pros and cons depending on the case. However, it is important to note that comparing only size 25 and size 35 instruments of the systems is one of the limitations of the current study. Moreover, only .04 tapered instruments of OneReci and single anatomy were used. Further laboratory and clinical studies will be needed to better analyse and comprehend the capabilities of OneReci.

## Conclusions

Within the parameters of the study, both size 25 single-file reciprocating systems appeared to be safe in preparation on moderately curved mesial roots of mandibular molars, with similar shaping performance and minimal apical transportation. However, larger apical preparation promoted significantly higher amounts of dentin removal, volume increase, and prepared root surface area in WOG. OneReci revealed promising shaping performance, principally compatible with the minimally invasive preparation approach, even with a larger instrument.

##  Supplemental Information

10.7717/peerj.15208/supp-1 Data S1 Raw data

## Additional Information and Declarations

### Data Availability

The following information was supplied regarding data availability: The raw data are available in the Supplemental File.
